# Making sense with numbers. Unravelling ethico‐psychological subjects in practices of self‐quantification

**DOI:** 10.1111/1467-9566.12894

**Published:** 2019-10-10

**Authors:** Jeannette Pols, Dick Willems, Margunn Aanestad

**Affiliations:** ^1^ Section of Medical Ethics Amsterdam University Medical Centre, and Department of Anthropology University of Amsterdam Amsterdam The Netherlands; ^2^ Section of Medical Ethics Amsterdam University Medical Centre University of Amsterdam Amsterdam The Netherlands; ^3^ Department of Informatics University of Oslo Oslo Norway

**Keywords:** ethics/bioethics, self help/care, self‐measurement, quantification, ethnography

## Abstract

Prevention enthusiasts show great optimism about the potential of health apps to modify peoples’ lifestyles through the tracking and quantification of behaviours and bodily signs. Critical sociologists warn for the disciplining effects of self‐tracking. In this paper we use an empirical ethics approach to study the characteristics and strivings of the various types of ‘ethico‐psychological subjects’ that emerge in practices of self‐quantification by analysing how people and numbers relate in three cases of self‐quantification: in prevention discourse, in testimonies from the quantified self (QS) movement and in empirical work we did with people with Diabetes type I and with ‘every day self‐trackers’. We show that a free subject that needs support to enact its will is crucial to understand the optimism about prevention. In the QS‐movement the concern is with a lack of objective and personalised knowledge about imperceptible processes in the body. These subjects are decentered and multiplied when we trace how numbers in their turn act to make sense of people in our empirical study. We conclude that there are many different types of ethico‐psychological subjects in practices of self‐tracking that need to be explored in order to establish what good these practices of self‐quantification might do.

## Introduction

There is great optimism about the potential gains of improving lifestyles as a means to prevent chronic diseases, and governments and to this end health care organisations have been encouraging the use of health apps that measure behaviour or bodily signs. In June 2014 the Dutch minister of health notified Parliament about her intentions to stimulate the use of health apps amongst the Dutch population, including her aim to have 75 per cent of elderly and chronically diseased people – if they want and are able – to use health apps in 2019 (VWS 2015). The idea behind this ambitious aim is that self‐tracking will lead to the prevention of disease and to better health for the trackers, and hence also to less costs for health care. These ambitions are propelled by statistical findings in population research that show that bad lifestyle habits, such as smoking, sedentariness and bad eating, cause more than half of common chronic diseases like diabetes and cardiovascular disorders (WHO 2008), and that these ‘lifestyle diseases’ are ‘modifiable’ (WHO 2016, 2008: 35). Lifestyles can be changed, assert prevention optimists, and this change is sought through state interventions such as agreements with the food industry and through stimulating individuals to manage their lives better. Crucial is the idea that people have a choice: it is possible for them to opt for a healthier lifestyle (Buyx and Prainsack 2012), and they may be better equipped (or ‘empowered’, see Constantini 2014, quoted in Owens and Cribb 2017) to do so through the use of health apps and the self‐measurements they enable (WHO 2011).

Dampening this optimism is the fact that prevention programs are often ineffective. If lifestyles are changeable, it is far from clear how this is best accomplished. There is a lot of quantitative research on the factors that shape the good and bad results of prevention programs. This research shows that long‐term interventions work better, and that programs should involve peer‐to‐peer contacts, address social norms rather than focus on education alone, and suggest healthy alternatives rather than attempt to prevent ‘bad behaviour’ (see e.g. Dusenbury and Falco 1995, Raczynski *et al*. 2012, Tobler 1992). However, such quantitative research into ‘health behaviour’, we argue with Cohn (2014), often loses track of the context in which ‘what people do’ has meaning for them. For prevention programs, for example, it makes a difference if people understand ‘eating too much’ as a celebration, as self‐therapy, as ‘too many calories’ or as a routine (de Laet 2017, Vogel 2018).^1^

In this paper we focus on the practice of using health apps in order to understand how self‐quantification shapes selves, and how selves shape self‐quantifications. We analyse assumptions prevalent in prevention discourse about how health apps might support individuals to change their behaviour through supporting their will. We then compare this analysis with expressions of the Quantified Self (QS) movement, and with our own research into practices of self‐quantification through the use of health apps. Our question is how people relate to numbers – and numbers to people – in practices of self‐quantification. What do self‐trackers say they want to achieve and how does this turn out? How do numbers ‘act back’? What does this imply for prevention programs that are based on the promise of health apps?

## Approach

Our approach of studying the shaping of selves through quantification in prevention practices is an alternative voice alongside a rather unison chorus of certain strands in the critical sociology of technology. To a greater and lesser degree, these critical sociologists highlight how (self‐tracking) devices *discipline* people into the regimes of the technologies they are using, a line of thinking that often evokes the notion of reflexive modernisation (Beck *et al*. 1994). Reflexive modernisation provides ‘a context in which subjects become increasingly individualised, introspective and responsible for the project of crafting their own self‐identities’ (Smith and Vonhethoff 2016: 8). The freedom of this modern subject is turned on its head: power mechanisms discipline people into shaping themselves as free and modern subjects. Lupton (2013) argues that individuals are made responsible for their own health, not as a newly gained freedom but as a duty that is imposed on them, even if it is happily embraced by the individuals pursuing it. Meanwhile, the ‘techno‐gaze’ offers ‘an unprecedented opportunity to monitor and measure individuals’ health‐related habits in a variety of milieus’ (Lupton 2012, in Smith and Vonhethoff 2016: 9, see also Rose 1999).

Although this argument is often well founded, our concern is that it imagines a too‐uniform (‘neoliberal’) subject. A determinist understanding of technology is too limited, and reflexive modernism is too broad a concept. The ways in which subjects are positioned in different settings and engage in modes of quantification, we argue, varies in important ways. It is in this play with freedom and power, determining and being determined, acting and being acted upon, that different forms of selves emerge, as a result of the relationships they seek to engage in and are engaged by (see Moser 2005). Here we use heuristics from empirical ethics to analyse how subjects emerge in practice, through relationships amongst people, devices and numbers (Mol 2010, Pols 2015; 2017, Sharon 2015, Swierstra 2013, Willems 2010, Willems and Pols 2010). Empirical ethics combines a ‘sociology of the good’ (Thévenot 2001, see also Boltanski and Thévenot 2006) and a material semiotic approach that does not ‘apply’ theoretical concepts, but studies what these concepts come to be – or how they become enacted – in specific contexts. What is ‘good’, is hence an open category that needs to be substantiated through empirical study. Strictly speaking it is not a fixed methodology, but a way to study how and where particular objects – or subjects, or concepts – come into being. Our question here is about the relationships between people and their devices for self‐quantification, to lean about the specific subjects that emerge.

Empirical ethics traces the intertwinement of what *is* and what is *good*. Specifically, we decipher the ‘ethico‐psychological selves’ that are enacted through self‐tracking. We unravel how such subjects are framed or enacted as having a particular (biological, sociological, psychological) way of being (a drive to chase self‐interests or to reproduce, having a free will, for example), while relating to the good in particular way (say, maximising pleasure or gain, reproducing genetic material, or doing the right thing). Ethico‐psychological subjects hence are described in terms of how they are made to act as well as how they make themselves act. As subjects, they are both object and agent: they are an object of study in the life – and behavioural sciences, but they are also understood as actively shaping and reflecting on themselves (as in the humanities). Psychology and ethics hence ask related questions. Why do people act as they do? The repertoires for answering them, however, differ vastly. Our empirical ethics approach brings these repertoires together by tracing particular intertwinements of facts and values in order to ask how self‐tracking incorporates particular understandings of the world as well as particular ‘forms of the good’ (Thévenot 2001). As we will show, devices and numbers also show this intertwinement of being and valuing.

In part I and II of the analysis, we explore how people make sense of numbers in three cases. The first case is a paper that reports on prevention ideals form the health app industry. We analyse Schüll's (2016) exemplary text, in which the will of the individual is a central moral and psychological category. The second case is formed by reports from the QS movement, to show how these avant‐garde number crunchers use data from self measurements to make sense of themselves as subjects. In this second part we add examples from cases from our interviews and observations with two groups of people who make sense of numbers: those we call ‘everyday self‐trackers’, or people who do not have health problems but measure themselves for different reasons (fitness, weight‐loss, out of curiosity) and people with type 1 diabetes, who measure and manage their blood glucose levels and have long experience in doing so. Within each group, 10 in‐depth interviews were conducted by two master's‐degree students, Liam Levy‐Philipp (2017) and Floor Visser (2017). Visser and Levy‐Phillip asked about the use and meaning of measuring devices and observed their use when possible. From the testimonies of the QS and the interviews, we identify two distinct styles of self‐tracking. The first is the objectivist‐changer style, which is oriented towards changing behaviour or outcomes based on objective measurements. The second is the semiotic‐aesthetic style, which is oriented towards discovering unknown patterns in behaviour, events or bodily signs.

In part III of the analysis we use our material from the everyday self‐trackers and people with diabetes to analyse *how devices and modes of quantification make sense of people*. People may put devices and quantifications to their own use, yet numbers also ‘act back’, leading to effects that were out of the control of the users. This became most obvious in our interviews, as testimonies of the QS movement generally narrate individual victories and measurements that have been ‘tamed’ in particular ways. The activity of numbers becomes most obvious when it leads to unexpected effects or frictions in people's lives. The styles of the self‐trackers are complemented by ways in which one may *become tracked* through self‐quantification. In our concluding remarks, reflecting on the recommendation of the Dutch health minister, we raise some ‘ethico‐psychological’ issues that might disturb an all too optimistic policy.

## Self‐tracking in a prevention logic: the importance of the will

To understand the logic of prevention – how people may change their lifestyles to prevent disease – we take up Schüll's (2016) thoughtful analysis of discussions in the app development industry. This is obviously a particular reading of prevention logic, but it is an interesting one to show how ethics and psychology may be aligned in discourses that stimulate prevention. Schüll argues that self‐tracking devices are scripted by the premise that people have to constantly decide what to do. The well‐being of these app users depends on the choices they make, but they are not very good at making choices. They never do the right thing, as they do not have the means to make good decisions. They tend to do what they do out of habit, lack of reflectiveness or laziness. Their intuitions, senses and automatisms are not well geared to attain what they *actually* want to achieve. They need support for this: a device that reminds them of their goals and hence ‘nudges’ them in better directions. Schüll (2016: 12) writes: ‘The nudge is a curious mechanism, as it both presupposes and pushes against freedom; it assumes a choosing subject, but one who is constitutionally ill equipped to make rational, healthy choices’. The will of the subject here is clear and well‐focused: people *want* to make healthy choices, but the *effectuation* of these choices needs improvement and outside support.

Schüll compares these ethico‐psychological subjects to entrepreneurs: they need information to make better choices, and tools to provide this information. They need to see patterns over time, like correlations between the flowing inputs and outputs of stock markets that inform one's business better. And this, she argues, is what these apps provide. They support users with making better decisions by ‘nudging’ (rather than forcing) them in the right direction through providing them relevant information in a timely manner. Nudging, here, creates awareness, as it interrupts unreflexive, routinised or intuitive ways of acting. The app suggests the better option, what the user would prefer to do if she were not distracted by routines.

In Schüll's analysis, the goals of the users are the same as the goals of the devices, and this is crucial for their good use: they provide tools for self‐induced nudging into self‐prioritised activities. They help us do what we *really* want to do. Although users might have a tendency to do the wrong thing, they want and are able to follow good advice to reach long‐term goals, even if these goals are in tension with immediate desires or habits.^2^ The rationality of these users is that they are able to act in accordance with the goal set by the device (and hence the user).

Schüll is not explicit about the status of her analysis: is it a description, a way of theorising health apps, or a proposal for normatively understanding its actual workings? We take it to represent an understanding of the *ideal use and user* of these apps that fits with a logic of prevention and concerns of the app developers. There are no frictions or ethical concerns if people use apps to achieve goals they set themselves. Such an understanding steers clear of the ‘bads’ of manipulation, disciplining or coercion into goals set by governments or industry, which is the central concern in the ethical discussions around nudging (see in this context: Owens and Cribb 2017). In Schüll's analysis of this self‐tracking discourse, it is up to the individual to decide which goals to pursue.

The self of prevention logic emerges: ethico‐psychological subjects are beings that may set themselves goals that are *their own,* consciously chosen goals. Once they have set their goals, they will also nudge themselves into achieving these. In this sense the individuals are *rational*; they strive for what they know or have decided they want to achieve. This implies that individuals may control the achievement of their goals – with a little help from their apps. They do this by an exertion of their will (they *want* to achieve the goals set), supported by self‐set nudges built into the apps, which are tools to help enact that will. In this vision, the use of apps for self‐measurement is *empowering* the individual to be enabled to better live up to self‐chosen goals.

Because individuals are free to set their own goals through their rationality and will, goal‐setting becomes an enactment of autonomy: it happens and has to happen outside of power relations. If force or pressure were used, the aura of will and rationality surrounding enterprise of self‐tracking would disappear. Nudges would become directives to follow goals set by others, raising again the concerns of critical sociology and ethics. Where support and encouragement are possible, force will not do, nor is surveillance an acceptable means. The mechanism to change lifestyles for prevention, both because of how people are understood to act as well as from an ethical perspective, is based on individual will, however fallible.

## Making sense of numbers

So now we have an understanding of the assumptions underlying an ideal ‘prevention self’. Now we will turn to the question what practices of self‐quantification look like and how ethico‐psychological subjects position themselves in these practices by actively making sense of numbers (see also Ajana 2017). The QS movement provides a very specific avant garde practice of self‐quantification. It is a popular topic amongst researchers because of this frontline position, and because self‐quantifiers are very open about their practices. They post their results on the internet and organise large meetings to discuss results.

Building on the analysis of Sharon (2015) and Sharon and Zandbergen (2016), we discerned two styles of self‐tracking in the texts of the QS movement, which may diverge or become linked in different ways. The first we call the ‘objectivist‐changer’ style of self‐tracking, which foregrounds an ideal of knowing the self through numbers. The aim is to produce objective knowledge about oneself, knowledge that is not disrupted by fallible perceptions or direct experience of the self (Sharon 2015, Sharon and Zandbergen 2016, Swan 2013).^3^ These ethico‐psychological subjects analyse their data with the aim of changing and optimising their bodies and health, by attempting to predict and control bodily processes and to act on this knowledge.^4^

The second style of self‐tracking we call the ‘aesthetic‐semiotic’ style. It betrays the roots of the QS movement in California, the breeding‐ground for hippies and others averse to authority (Sharon and Zandbergen 2016). It is an anti‐authoritarian orientation that experiments with different forms of self‐awareness. Knowledge of the self is seen in aesthetic terms, as creating different and interesting forms of self‐awareness. A different experience of the self can be a goal in itself. The artist Alberto Frigo, for example, photographs every object he takes in his right hand (ibid). This changes his understanding of himself, he writes, by providing ‘some kind of DNA code of my life’ (ibid: 6) through the collection of images of thousands of objects.

Aesthetic descriptions, however, may also be used to subvert dominant ways of knowing that prescribe what is ‘normal’ or ‘common’ to experience, often challenging medical or psychological modes of understanding and acting. Both tracking styles come together when these ethico‐psychological subjects develop *n* = 1 experiments which are tests with one subject, here: the person conducting the experiment. They do this to gain knowledge about themselves with the aim to improve medical knowledge. They boast that, with this personalised knowledge, they will outsmart their doctors: they become the experts on their own bodies. Sharon (2015: 18) refers to Larry Smarr who diagnosed his Crohn's disease through self‐measurements before his doctors were able to reach the same conclusion.

For the ‘objectivist‐changers’, quantifications can make the body predictable, much in line with the understandings of behavioural economy. If one thinks of one's body weight as the result of the energy taken in and the energy used, for example, it becomes possible to calculate a program on how to lose weight. The body becomes calculable and predictable, or at least, that is the aim. One of our interviewees, George, a person with type 1 diabetes, exemplifies this calculability. He explained how he measures a variety of variables (blood glucose, insulin, HbAc1, calorie intake, sleep, weight, exercise, environmental temperature) with a variety of technologies (four apps that do not synchronise, an insulin pump, a sensor, his smartphone) in order to predict his blood glucose levels and influence them. George used these data as well as the continuous measurements of his blood glucose provided by a sensor. The sensor is necessary for making such measurements possible, but is too costly to be used by many.

Rather than attempting to stay within a certain range of high and low glucose levels (which would mimic ‘average’ glucose metabolism for non‐diabetics, and is generally the aim for people with type 1 diabetes), he aimed for a *flat* line by strategically administering insulin and food at times when his blood glucose was going up or down. He explained,

I inject insulin in the morning about 1 hour before I eat, because I have noticed that it starts to work after 1 hour. Then I see a small dent in my glucose graph, so that is when I start to eat.

By considering and calculating different variables, George could design new and clear targets. With his extensive measurements, he made the behaviour of his body predictable and controllable.

The ‘semiotic aesthetes’, on the other hand, are not concerned with predictability, nor with the changing of their own behaviour or bodies for the better (hence there are no type 1 diabetes subjects in this group). In our interviews, these trackers demonstrated a clear preference for recording certain variables out of interest rather than to propel change. It worked rather more like a diary than a log, and events could be traced back to what they had experienced in real life. Here is an interviewee and every‐day self tracker from the semiotic‐aesthetic style, Siena. She explains how she used self‐tracking:

Well, I don't look at it [the graphs from the health app] every day. But I did learn a lot from it. It gave me the amount of calories that I was using while I was biking, for instance. So it's interesting to see what's happening if the wind is blowing from the north or the south, if it takes a lot more calories. Then I have to bike against the wind. That was all new, so I think I am quite interested in the information that it gives me. It's not that I want to know how many calories I burnt during the whole day, but rather what is happening now, or see what happened then, with my heart rate … and, oh yes, there [points at a graph] I was working in the garden. Ha ha, yes, we were cutting a tree, quite a big tree, and suddenly one of the very big branches fell down instead of going slowly down the rope. So then in the evening I looked and saw my heart rate … ‘What happened? 180!’ That was exactly at that time! [Laughs]

Siena did not use her measurements to calculate calorie intake, but to learn about the influence of the winds and falling branches on her body. The app's graphs illustrate – and interpret – her body's experience of the impact of the falling branch. Data are a way to learn how the body lives events in its own way.

The semiotic aesthetes also used results from their self‐tracking to criticise existing norms. Sharon (2015: 18) describes Dana Greenfield's project about how she mourned her mother after she passed away. Greenfield did not want to mourn ‘by the book’, and instead registered how often she thought about her mother, what triggered that, what mood she was in at that time, and so on. In this way she developed an individual map and picture of how her mourning evolved. This was an alternative for living up to standards on how mourning should be done, when ‘normal mourning’ should be finished and how intense it should be. In this way she made self‐measuring a constructive act to *design* mourning in a new, personalised way, providing new accents and individualised norms for how mourning is and may be done. It is a creative process rather than one aimed at controlling variables.

The mourning example shows a clear link between what is tracked, counted and archived and the ways people make sense of it. Possible modes of experience are developed through the experiment. In this style, self‐tracking is not aimed at *changing* what one does, but about carving out individual pathways to *understand* what one does. This is simultaneously a critical epistemological statement on popular modes of quantification in the social and medical sciences. Calculating average probabilities across defined populations creates a norm that is not directly applicable to individuals. The mode of quantification these self‐trackers put forward, however, is based on personalised calculations, and they produce a type of knowledge that is specific to the individual. It can be made relevant for others who may repeat the experiment themselves. It is a suggestion for *doing science* differently, and it shows that different modes of quantification allow for different frameworks to interpret numbers.

Another semiotic‐aesthetic example can be seen in the blog by Nancy Dougherty, who registered how often she smiled in a day (Davis 2013, see also Sharon 2015: 23). Dougherty recorded a higher smile frequency when colleagues approached her, which she interpreted as her *liking* her colleagues more than she had been aware of. That her smiles represented an unacknowledged affection is a highly original interpretation, made by relating numbers to feelings. Quantifications have to be made sense of in order to be useful or mean anything at all.^5^ Dougherty's conclusions are quite remote from a scientific dream of objectivity aimed at excluding the subject doing the measuring. Even when measurements exclude influences from the subject doing the measuring, interpretative frameworks are always needed to make sense of the outcomes, including when these numbers are collected without any particular hypothesis.^6^

### Qualifying subjects

Even though the cases show an inseparable link between numbers and interpretations, the QS movement does not foreground an interpreting or ‘qualified self’ who learns about itself through direct experience. The qualified self serves as a somewhat caricaturised backdrop of imperfection, against which quantifications get their value and shine. Phenomenological tools such as sensing, feeling, memory and introspection tie the qualifying self to the here and now, in contrast to the temporalities that may be created by quantification over time. Qualified perceptions are thought of as fallible and limited, and are to be improved and complemented by quantifying the self. In contrast to the ethico‐psychological subject of prevention, here it is not the *will* that these self‐trackers see as in need of support, but rather their *possibilities for knowing themselves* and what they or their bodies do. The senses are insufficient tools, too limited to adequately keep track; nor are the generalised quantifications of the sciences acceptable, as they are not specific to the individual.

If what people experience and what they are aware of is only a limited part of what informs their activities, measurements (of smiles, of glucose levels) can be a way to feed self‐awareness, giving insight into what escapes everyday perceptions. There is not a ‘meaningful’ self, as in psychoanalysis, where turbulence is created by unconscious desires and emotions pushing and wrestling within the psyche for expression. These need extensive interpretation as a way to liberate oneself of their consequences. Self‐trackers, however, battle with a more prosaic unconsciousness of bodily facts, habits, automatisms and metabolisms of which the subject is not aware. Wolf, a prominent person in the QS movement, writes:

When we quantify ourselves, there isn't the imperative to see through our daily existence into a truth buried at a deeper level. Instead, the self of our most trivial thoughts and actions, the self that, without technical help, we might barely notice or recall, is understood as the self we ought to get to know. (Wolf 2010, quoted in Sharon 2015: 12)

Rather than representing unresolved conflicts through interpretation, these unconscious ways of acting represent physical and mental processes and events that are not under the control of the individual when they pass unnoticed. These processes simply ‘happen’ and do so well away from the experience of the subject, while shaping the subject through its automatic physical and physiological responses.

For self‐quantifiers, measurements are ways to trap unconscious processes; objectivist‐changers hope, too, to tame these automatisms. The visualisation and analysis of measurements over time makes unconscious habits and processes ready for reflection and, again for the objectivists, adaptation. Self‐tracking and quantification allow one to learn about oneself by bringing to light these automatic, unnoticed processes.

Interestingly, the tracking and analysing of different ways in which they are physiologically determined as subjects, enables the ethico‐psychological subject of the QS movement to insert free will in a new way. Individuals may influence their unconscious behaviours and processes, if they prudently quantify and actively manage them. By mastering different forms of being determined by one's bodily processes, space is created for agency, meaning and morality, for example to achieve a flat line on a blood‐sugar graph or to observe one's own personal style of mourning. By gaining a peek into what our bodies do outside our awareness, we may find ways to control them, not by nudging ourselves towards self‐chosen goals, but by getting to know the ‘ways of the body’, manipulating these, or understanding them better. It is not the will that needs support, but one's knowledge needs to be improved. The quantified and autonomously acting body becomes ‘one's own’ again.

## Numbers make sense of people

In the examples above the relation between numbers, or styles of quantification, and sense‐making has a clear direction: individuals, as ethico‐psychological subjects, interpret numbers that represent their beings and doings. Numbers *in themselves* are not objective representatives of truth, even if they are taken to be just that. Numbers are gathered and interpreted within particular styles, sets of values and meanings (art, knowledge, activism, health, etc.). This semiotic activity, however, also works in the opposite direction: *numbers make sense of people* and act upon them. Willems (2000) describes how, through repeated measurements, his informants with asthma came to learn to sense these quantifications, and how this changed their way of experiencing their bodies by their ‘incorporating’ their measurements. This also happened to some of our informants with type 1 diabetes, if their insulin metabolism was regular enough. A certain number became linked to a certain feeling that signified (too) high or low blood sugar levels. People learned to train and educate themselves and what they felt by making use of numbers.

Below, we discuss how numbers make sense of people by: (i) specifying their goals, (ii) intensifying particular concerns, (iii) suggesting coherence and predictability, and by (iv) moralising lives under the guise of ‘fact collection’. We also briefly discuss a fifth way: numbers make selves transportable through their data, allowing for their re‐interpretation in new contexts, but a full discussion of this remains out of the scope of this paper. Our analysis foregrounds our interviewees who enact the objectivist‐changer style, as change is most visible here.

### Specification: devices and quantifications translate goals

One of our informants with type 1 diabetes, Jan, told us how his experience changed when he had to quantify things he had never dreamt of quantifying before:

I went to the dietician the first time, and she asked: ‘How much do you eat?’ How would I know that! I eat until I have had enough. So that was very difficult for me. […] And what happened last time? There was a day when I had injected [insulin] and then the potatoes were burnt. And you don't realise, and you are sitting on the couch [that evening] and then, erm … [your blood glucose level is all wrong]. And same with sports. If I plan to go out for a run in the evening, and I think later: ‘Oh, I don't feel like it today’, then I do not go. But I then I would have to inject more.

In this interview, Jan described how he calculated how many potatoes he had eaten, and their size, and how much insulin he would need to balance that amount out. He thus had to shift from a qualifying perception (‘I have eaten enough’) to making a set of quantifications. This shows how types of devices and ways of quantifying translate and hence re‐interpret people's goals and self‐understanding. To live healthily or to optimise one's fitness becomes a very specific target, involving, say, so many calories or exercise and so much insulin. Devices and quantifications specify general goals like fitness and health.^7^

Numbers do not come to us in an abstract form: they are produced through devices or apps that turn events into numbers. Understanding how these devices work is crucial for understanding self‐quantification and concomitant different ways of living. A very clear example can be seen in the ‘traditional’ blood sugar measuring pen, with which measurements are taken at particular times in the day (usually 4–6 times). These discrete measurements often serve as feedback for the very short term and no particular relationship can be made between them. Our informants who wanted to minimise the impact of diabetes on their lives, including the impact from monitoring blood sugar, preferred this way of measuring (Visser 2017).

In contrast, the sensor's continuous monitoring puts measurements in relation to one another, allowing one to see trends and to relate spikes or crashes to an event or other variable that might happen to influence blood glucose levels. This was a good method of tracking for people, like George above, who wanted – and could – exert a firmer control over their diabetes. But such continuous measurements involve another set of quantifications: sensors are costly, and only paid for by Dutch health insurance for people whose blood sugar levels are hard to tame. For George, the sophisticated combination of measurements made his bloodsugar levels predictable – and their measurement prominent in his life. Even his diabetes nurse consults him as an expert when she is confronted with difficult cases.

The type of device one uses thus structures a specific form of quantification, which then opens up a specific repertoire of activities to pursue. We saw how individuals negotiated and adjusted their goals in relation to the specific affordances of the technologies they used. Melanie, for instance, wanted to stay fit and healthy:

I think that's the problem with focusing on weight loss. Because if I am exercising regularly and eating well, then I feel okay. That's why I don't like making weight loss a goal. Because if I say: ‘I want to be this number by a certain time’, then I feel a panic. And there shouldn't be panic. I should be enjoying things. And that's why I don't make a number goal. I have a number in my head, but I don't write it down, and I don't put all my focus on it. So, it is my goal, my motive is to lose weight, but I just think that for overall mental health, there needs to be more than that.

Melanie had to invoke the category of ‘mental health’ to make her general goal of ‘health’ feasible, which had failed when ‘health’ was specified as ‘losing weight’. In interaction with their devices, our informants translated and kept adjusting goals in order to forge a liveable practice. Specific goals that seemed rational on the drawing board had to be fitted in with other concerns in daily life – if only to fit in the nonspecific goal of living an enjoyable life, which had not been part of Melanie's first calculations regarding weight loss. Older people with type 1 diabetes also had seen specific goals for blood sugar regulation come and go with the medical fashions of the day, moving from very strict towards to more lenient regimes.

Self‐trackers reported disappointment in what goals their health apps could achieve through their specification. Gerard lamented:

There are a whole lot of things that it [the self‐tracking device] is tracking but not really a whole lot of things that give proper insights. It tracks so much, but at the same time it does nothing. So, I think that is a bit of the issue that I have with it. It can do a whole lot and I don't think that the algorithms are that hard to make. Take my heart rate. It doesn't show me that I have a higher heart rate these past months or if there is a difference in months that I work out, for instance, that my resting heart rate is lower for example, or anything like that. That is data that would probably motivate people more, into actually doing [work‐outs] … But yeah, that's not available. For me it was like: well, I saw the gadget and thought: ‘I want that!’ Ha ha!

This aesthetic tracker's love of gadgets made him use the devices, not – or no longer – propelled by the hope that these might provide him with information to optimise his fitness, but simply because he enjoyed having them around.

### Intensifying concerns

Measuring could lead to an intensification of the relevance of particular concerns in life over others. ‘Becoming obsessed with numbers’, as some put it, was a concern that was often articulated by our informants. Johanna described how the feeling of control the measurements provided could turn against her: ‘In high school I was just doing [tracking] the steps, weight and water’ . Liam, who was interviewing Johanna, prompted her to continue: ‘And the reason you stopped was because …’ Johanna explained:

Well, I felt like it was fun in the beginning. I dunno, it's like: ‘Oh it's new!’ and then you lose concentration. And afterwards, in college I was just like: ‘I don't feel like being this controlling’, kind of. It felt like controlling after a while. I was like: ‘I hate tracking everything!’ It felt like I was controlling my entire life because I was tracking everything, and it's all numbers. And I was like: ‘This is just too structured, and I feel like I am living day to day’. It got really like: ‘I wake up, I drink this much water, I weigh this much, and I exercise …’ It felt too routine, I guess.

The demands of collecting numbers could become so intense that it could seem like the only relevant thing to do. There was no pleasure, there were no surprises or unexpected events, just the routines of diligently recording and living up to one's numbers. This Johanna could not uphold. She even suggested the roles were reversed at a certain point (see also Dudhwala 2018):

Maybe I would say that it [self‐tracking] gives you a little bit of control over your life. You know, life has become so complex and busy, that sometimes it gives you the feeling of stability. But it can also have the other effect, like vice versa, it gives you the feeling of stress when you look into it too much. It's difficult to explain but I got it [fitbit] because I wanted to be more in control. But sometimes I feel that I am less in control because of it. That it has taken over my life.

Specification and the selectivity that comes with it worked to intensify concerns about particular variables. Some things become more important than others. Piras and Miele (2017) show that pregnant women with type 1 diabetes struggled with an intensified regime to control their blood sugar levels, to protect their unborn children. Their concerns were about the ‘other things in life’ that mattered to them, which had to be sacrificed for intensive monitoring. They missed the freedom to improvise, for instance, to enjoy the type of meal one fancies when going out with a friend. They complained about standardised lifestyles and experienced the regime as uncaring. Tellingly, one woman said the monitoring and prescriptions made her feel sick rather than healthy. The specific aim of managing their diabetes could become so intense as to turn into an activity to live for, not a condition that allowed for doing other things in life.

### Suggesting coherence and predictability in a lifeless calculable

This ‘metrification’ (Yates‐Doerr 2015) or quantification of life comes with the suggestion that there is a calculable and controllable way of achieving a goal. Taking in X calories while expending Y calories through exercise, for example, would result in weight loss if Y is larger than X. We saw some successful and troubled examples of this above. But this predictability did not always come to be, which caused a lot of frustration. Our informants with type 1 diabetes described their frustrations and despair when they could not link their measured blood glucose levels to their experience or to calculations of food intake and energy use (see also Hortensius *et al*. 2012). If measurements are never what they should rationally be, they provide no tools to learn from and give no clues for acting. Melanie explained how this lack of reliable results from dieting spurred more binge eating:

It's kind of a weird game. Because if I cheat, or if I go out drinking all weekend, and I don't see any weight loss, I think: ‘Well I expected that!’ But if I am working really hard [and there's no loss], then it sucks. If I have been working out and eating well and my weight doesn't change, then that feels really bad. And that's when I start binging. I have gotten better, but I used to binge when I would behave really well all week and I didn't lose weight.

The examples show the despair that rises up when one's body does not behave in predictable ways. It challenges the very rationale for ‘behaving well’, however, without completely overthrowing its truth. In Melanie's case, the resulting binge was not liberating or based on an alternative hypothesis, but still felt as ‘bad behaviour’.

Quantification often fails, in more or less dramatic ways. Our informants teach us that ‘living’ is an open‐ended process in time. The potatoes may get burnt. Things may change for no understandable reason at all, as Jan described:

For years I've injected 14 units in the morning, with a slice of bread and a glass of milk. For years. Perhaps for 10 years. And now I inject eight or nine units in the morning with the same food. Why is that! Tell me, because I don't know! And there is not much you can do about that. You can use apps and keep track of all sorts of things, but I've used 14 units for years and that used to be right. But now …

In the end, the perfect calculation can never be carried out ‐the future can never be seamlessly predicted.

### Moralisation through facts

As we saw in the objectivist‐changer style exemplified by the QS representatives, numbers have a strong appeal of showing ‘what is really there’. Measurements present people with facts about their bodies. Unless the devices are broken or imprecise, one thousand steps have indeed been taken, a blood‐sugar level is 6.1, one's weight is 52 kilos and 300 g. But our informants were very clear about this: numbers are not only factual, they are also normative: they are good or bad. Numbers enforce certain ideas of the good. If the numbers are bad despair may well follow (‘nothing can be done about it’), as well as feelings of being punished (‘you did not behave well’). One informant told us that she set her step‐counter target in such a way that she would get a daily reward rather than a punishment (‘at least one thing achieved today!’). But bad numbers could also lead to unwanted moralising from others: the tracker is being tracked, just as the pregnant women felt overly controlled by their doctors. Sara recounted how she worked to avoid that critical gaze:

You go to this doctor and he sees your readings and says: ‘Well, you must be careful, this should be lower’. And then you get the entire lecture. Then I think: ‘*You* should try paying attention to this 24 hours a day. I am not a machine! […] I *know* it is wrong. At a certain point I'd just time my glucose readings in such a way that it would not result in criticism anymore.

Notice the machine metaphor, pointing to the specification, intensification and rationalisation of goals, as well as to the doctor's insensitivity to the burden of moralising through numbers. In self‐tracking aimed at change, the normative and the factual are intertwined.

Devices are programmed to direct how a certain good may be achieved. Schwennesen (2017) describes a case in which a woman used a device at home that gave physio‐therapeutic instructions for patients recovering after a hip replacement. It was a very sophisticated device that worked with sensors, to provide feedback on motions in space (‘Lift your knee higher!’). The patients could exercise at home, so they did not have to come to the clinic for rehabilitation. In this case, by sticking to the protocol of the device, the woman actually over‐trained, damaged the new hip and spurred a need to replace the other hip. The clinicians had aimed to implement the good of home rehabilitation, which their patient followed. She took the instructions as the right ones to follow, but these turned out to be too unspecific, standardised and harmful. The ideal user here would have to be able to modify the device's instructions.

### Making selves transportable and re‐interpreting them

By generating sets of numbers, self‐tracking individuals create data about themselves or what Ruckenstein (2014) calls ‘data doubles’. These data doubles are the look‐alikes of the selves collecting them, a snapshot presented through numbers. But, as we have seen above, these doubles are not exactly identical twins. They are selves translated into something else, selves turned into numbers that specify, intensify, moralise and attempt to make predictable. *Ceci n'est pas une pipe!* An object does not equal its representation.

One effect of translating selves, events or activities into numbers is that numbers make individuals or their data look‐alikes transportable. As particular specifications and recordings of the individual, these data can travel to other places. This means that an individual's numbers may become part of different networks, where they are fit into new relationships that foreground different concerns from the ones for which the numbers were originally collected. This is what Nissenbaum (2010) points out when she shows that we experience concerns with privacy when our data travel and are used in contexts that do not seem proper. Doctors may ask us all kinds of questions, but we would feel our privacy betrayed should those answers be published on the internet. By travelling, both the factuality and normativity of numbers change (see also Mittelstadt and Floridi 2016: 321).

One reflexive user of a step‐counter noted on his blog and in an interview with us that if he did not reach the set amount of steps each day, he would shake his mobile phone in order to reach and record the right amount of steps. Indeed, self‐tracking here did not serve to *objectify* behaviour. The user was concerned, rather, that measurements would solidify as ‘evidence’ of what he had done. This evidence could travel unmediated by his explanation of it. It could be stored in databases and become unchangeable, true. Our phone shaker explained in an interview that he did not want to be recorded as a lazy person or someone with a weak mind who cannot even reach the simple goals he has set for himself.

What becomes of these data look‐alikes? They change context and depart from the goals that had directed their collection and how they were made sense of. They may not even resemble the selves they are thought to represent anymore. Here's Brittany from South Africa, who got an Apple watch from her health insurance company. If she earns her points through meeting her exercise targets, the watch is free. She couldn't afford to buy it herself. And she wanted it! She explained:

At some point I wrote an exercise schedule on my phone because I know I have to get 900 points. I have to push during the weekend, so should something happen in the week then at least I am sitting at 600 or so. It's quite important to get 900 points. So I plan. Planning is key, it's a big part of it.

Brittany gets her points, not to become healthy (is it actually wise to do all your training in the weekend?) but to obtain the watch. This has implications for how her data may or may not indicate anything about achieving health though exercise – though it does show that some people can be pushed into exercising when given a chance to own something they want, even if it is unrelated to health goals. The insurance and the user have different goals, and the insurance uses the watch to force users into the goals it finds important. The unreliability of decontextualised numbers is a concern for the validity of the knowledge created from them (Aicardi *et al*. 2016, Mittelstadt and Floridi 2016), and often very different goals need to be juggled to collect data at all (Tempini 2015).

## Conclusion

What does our empirical ethics approach learn about the characteristics of psychological subjects as well as about their strivings? An obvious conclusion is that there were many different types of subjects. This nuances the extremely voluntarist ideas from prevention, as well as a technological determinist idea of the subject from critical sociology of technology. The different ways of understanding what a subject is, as well as what it wants, showed different relationships between people and numbers. We started with discussing the prevention discourse, where free will reigns, along with the discretion of self‐quantifying ethico‐psychological subjects to set their own, self‐chosen goals. In this conception, one's will may be strong, but its execution needs support. Nudges from health apps could provide this support, as a reminder to individuals about what they really want. We found that objectivist‐changers shifted the diagnosis of the problem to one in which self‐quantification is an answer. The problem was not a weak will, but a lack of knowledge about processes in a body that lives according to its own rules and metabolisms, of which people are not aware. Free will remained crucial, as these unconscious processes could either be controlled or could be given pride of place to correct social or medical norms with personalised ones. Apps served as instruments to reveal hidden mechanisms that purposeful individuals could use to their benefit.

In our interviews about practices of self‐tracking from people with type 1 diabetes and from everyday self‐trackers, individual will was much more fragile. It was but one of the elements in the manifold, shifting relationships that influenced ‘what people do’ in their practices of self‐quantification. A lack of knowledge that could be undone by learning about the body turned out to be complex. Modes of quantification inserted coherent, calculable and hence controllable repertoires for action into people's lives. These specified forms of the good to achieve given a specified diagnosis of the problem that intensified the search for solutions. Broad aims like health and well‐being were translated into so many steps a day, or so much weight loss each week. People actively engaged with these coherences, by living up to them, by resisting them for other goals, by re‐defining and adding new goals, by replacing them with new regimes, or by ambiguously accepting and rejecting them. They struggled with incalculable bodies and situations, and clashing – or failing – frameworks for interpreting these.

What people did and how was contingent on relationships amongst people, devices and contexts – of medical prescriptions, ideal body weight or aesthetic curiosity. Interpretations could be out of their influence, as when their data travelled to places unknown to their collectors. The crafting, breaking and reframing of relationships were an open‐ended process. People got bored with their devices, invented new ways for how to live with them or subverted them, took time‐outs or sought out fruitful combinations of health apps. There was no clear or certain line between self‐quantification and health, a relationship that could also fail miserably.

Our analysis showed how numbers cannot be separated from interpretations, as interpretations are built into the technologies and regimes used, and must be translated to specific situations. Numbers are collected for specific aims. The specifications, intensifications and coherences that quantifications brought came with an aura of objectivity, because numbers seemingly erased subjective elements and interpretations. In the context of self‐tracking practices, however, a strong appeal to truth in combination with clear directives on how to act made numbers double‐edged interpreters of life. Numbers have a strong morality within the socio‐material context in which numbers are qualified as adequate, true, boring, evidence of ‘bad behaviour’, or good or beautiful reporters on the wonders of the body. With our approach of empirical ethics we could show the different relations between facts and desires that are part of very different sets of self‐tracking practices. Indeed, the literature about self‐tracking from the social sciences that is avalanching at the moment, has important work to do to uncover many more types of ethico‐psychological subjects and workings of (self) quantification (for instance the work on affective aspects of technology use and sensory aspects of digital health, Lupton and Maslen 2018, Schwennesen and Koch 2009).

Is the Dutch health minister's advice a good one for improving health? People and apps turned out to be *different beings* than speculated on in prevention discourse. Self‐tracking practices were varied, and devices did not simply support an individual will to stay healthy. Apps translated goals and made people do different things than hoped for, even when striving for health or fitness. Care for health happened in relation to disease, social norms and medical regimes, which made peoples’ goals less ‘their own’ than prevention discourse would have it. Different from QS objective changer style ideas about collecting facts about a singularly interpretable body, self‐tracking practices produced multiple coherences, linking to different versions of understanding the body and what is good for it. Trackers could experience ‘wrong numbers’ as punishment, and this might keep the less athletic from using apps (see also Depper and Howe 2017). It is proved difficult to make apps produce *relevant* rather than merely *quantifiable* information.

Our analysis also showed that people *strived for* different things than hoped for, and that apps interpreted and translated these goals. In practice, health turned out to be an unspecific value that, when specified, emerged as just one, very specific goal between many others (enjoying life, good mental health, not being concerned by diabetes). Hence, self‐tracking could lead to unintended health hazards, such as obsessive concerns (too much specification) or overtraining (too little specification).

So there is no simple (calculable!) correlation between the use of health apps and a better lifestyle. Yet one problem policy makers might be able to tackle is the risk of unwanted data travel and de‐contextualisation, by supporting the production of ‘standalone’ apps that do not automatically send user data to companies. Rather than supporting the production of unreliable knowledge made from ill‐collected data, prevention aims would be better served by investing in the development of more contextualised studies of what people are doing when they are tracking themselves or work on their health in other ways, how this interprets the aim of supporting healthy lifestyles, and how individual an population health may best be synchronised accordingly.

## Notes

1 Vogel and Mol (2014: 306) ironically state that if behaviour is the target, the practical achievement would be to make people ‘behave well’ and ‘be good’ (315), rather than learn how to eat well.

2 H.G. Frankfurt (1988: 128) speaks of primary and secondary desires: ‘Besides wanting and choosing and being moved to do this or that, man also may have desires to have (or not to have) certain desires and motives. They are capable of wanting to be different, in their preferences and purposes, from what they are’.

3 Daston and Galison (2007: 17) describe the historical emergence of objective knowledge in relation to a simultaneous downplaying of subjective knowledge and perceptions, holding up knowledge that does not leave traces of the knower. Rather than relying on phenomenological tools or experience, quantification through self‐tracking can teach people things about the body they cannot know through subjective means. This connection of numbers to objectivity and truth to numbers is crucial.

4 This orientation towards change is also an aim for people who track themselves to make medical interventions in a third pattern that we call the ‘planner’ style. We discuss this elsewhere, as they are not quantifying their outcomes but planning what they need to do. This seems to be a more hopeful way to target ‘good health behaviour’ than health apps, as it is characterised by action rather than analysis (see Levy‐Philipp 2017).

5 One could of course add that selecting the hypothesis, the instruments and the concepts is also a highly interpretive way of acting. This is however not the common understanding of quantitative research, where good methods exclude the influence of the subject.

6 This mechanism may explain how pharmaceutical industries can simultaneously use state‐of‐the‐art methods, while still presenting results in a too‐rosy fashion. Methods cannot guarantee objectivity all by themselves, and they also shape in a particular way what results will look like (see Law *et al*. 2001, Ruppert *et al*. 2013).

7 We don't use the word ‘reduction’, as this seems to imply that there is one ‘whole’ that is quantitatively lessened in one particular way. We'd rather point to the *different qualitative ways* in which specifications are made, which makes for different consistencies that do not ‘add up’ to a whole.
